# The effects of combined endurance and resistance exercise training in heart failure: a meta-analysis

**DOI:** 10.3389/fcvm.2026.1777036

**Published:** 2026-06-23

**Authors:** Shun Hu, Yue Xu

**Affiliations:** 1 Deparment of Vasculocardiology, Ezhou Central Hospital, Ezhou, Hubei, China; 2 Department of Respiratory and Critical Care Medicine, Ezhou Central Hospital, Ezhou, Hubei, China

**Keywords:** clinical efficacy, combined endurance, heart failure, meta-analysis, resistance exercise training

## Abstract

**Objective:**

To evaluate the efficacy of Combined endurance and resistance exercise training in patients with heart failure (HF) and provide an evidence-based foundation for clinical management.

**Methods:**

A comprehensive search was conducted across domestic and international databases from inception to November 2025 for studies on Combined endurance and resistance exercise training in HF. Study quality was evaluated using the Cochrane Risk of Bias tool. Literature management was performed using NoteExpress 3.2. Data collection and extraction were conducted with Excel 2003. Statistical analysis was performed using RevMan 5.4.1 software. Heterogeneity was determined via the Q-test (*P*-value). Accordingly, the pooled effect size was calculated as the mean difference (MD) or odds ratio (OR) using a fixed- or random-effects model. Forest plots were generated, and publication bias was assessed via funnel plots.

**Results:**

Nine studies met the inclusion criteria. Meta-analysis results demonstrated that, compared to the control group, the intervention group showed significantly greater improvement in peak oxygen consumption (MD = 4.36, 95% CI: 1.68–7.04, *P* = 0.001) and quality of life (MD = −9.32, 95% CI: −16.92 to −1.71, *P* = 0.02). Performance on the six-minute walk test was also significantly better in the intervention group (MD = 53.76, 95% CI: 38.81–68.70, *P* < 0.0001). However, no statistically significant differences were observed between the two groups regarding sleep parameters or depression levels. Sensitivity analysis indicated that the findings were stable and reliable. Funnel plot results suggest a relatively low possibility of publication bias.

**Conclusion:**

Combined endurance and resistance exercise training is beneficial for improving clinical outcomes in HF patients.

## Introduction

Chronic heart failure (CHF) is the end-stage of most cardiovascular disorders, characterized by progressive cardiac dysfunction, reduced cardiac output and systemic circulatory impairment (1, 2). Global epidemiological data show 6.5 million HF patients in the United States (projected to reach 8 million by 2030) (3) and an estimated 8.9 million in China as of 2019 (4). With the accelerating aging of the global population, the prevalence of CHF among the elderly is expected to rise further. CHF is marked by a protracted course, frequent exacerbations, and difficulty in achieving a complete cure, seriously impacting patients’ physical and mental health and quality of life (QoL), while significantly elevating the risks of rehospitalization and mortality (5, 6).

Pharmacological therapy remains the cornerstone of CHF management, but long-term use carries risks of adverse effects that may compromise adherence and prognosis (7, 8). In recent years, alongside the transition toward a holistic “biopsychosocial” medical model and the growing adoption of cardiac rehabilitation concepts, exercise rehabilitation, a core component of cardiac rehabilitation, has gained widespread recognition as an effective non-pharmaceutical intervention for improving functional status and prognosis in CHF patients (9).

Since the 1970s, accumulated medical evidence has supported the positive role of scientifically prescribed, moderate-intensity exercise in HF rehabilitation (10, 11). Progressive rehabilitation training, centered on aerobic and resistance exercise, is an established regimen (12). While research on aerobic exercise in CHF rehabilitation is relatively extensive and its clinical application is quite common, studies on resistance exercise remain comparatively limited, and its clinical implementation is not yet widespread (13). Evidence suggests that aerobic exercise enhances peak oxygen consumption (VO_2_), boosts cardiac output, and reduces body fat, thereby improving cardiopulmonary function (14). Resistance exercise more effectively enhances muscle strength and functional reserve, with milder heart rate elevation and preserved myocardial perfusion pressure, potentially protecting myocardial oxygen supply-demand balance (15).

However, existing studies on progressive rehabilitation training in HF show significant heterogeneity in sample size, baseline characteristics and intervention protocols, and most focus on single exercise modalities, failing to reflect clinical practice (16, 17).

Against this background, the combined aerobic-resistance exercise model under progressive rehabilitation training has theoretical and practical value, integrating central cardiopulmonary benefits of aerobic exercise and peripheral muscular benefits of resistance exercise. This study systematically meta-analyzes the effects of this combined model on key outcomes in HF patients, including cardiopulmonary function (peak VO₂), exercise tolerance (6-minute walk distance, 6MWD), QoL and rehospitalization rates, to provide high-level evidence for its standardized clinical application and personalized optimization.

## Materials and methods

### Literature search

Computerized searches were performed across several authoritative medical and biomedical literature databases, including PubMed, Embase, Web of Science, and the Cochrane Library. The search was limited to English-language publications, with data updated through November 30, 2025.

### Search terms

Keywords: Heart Failure, Cardiac Rehabilitation, Exercise Therapy, Resistance Training, Aerobic Exercise.

Free-text words: “heart failure”, “cardiac insufficiency”, “CHF”, “cardiac rehab”, “heart rehab”, “exercise train”, “physical train” OR “exercise program”, “resistance train” OR “strength train”, “weight train”, “aerobic train”, “endurance train”, “cardiovascular exercise”.

### Search strategy

(“Heart Failure” [MeSH] OR “heart failure” [tiab] OR “CHF” [tiab]) AND (“Exercise Therapy” [MeSH] OR “exercise train” [tiab] OR “physical train” [tiab]) AND (“Resistance Training” [MeSH] OR “resistance train” [tiab] OR “strength train” [tiab]) AND (“Aerobic Exercise” [MeSH] OR “aerobic train” [tiab] OR “endurance train” [tiab]) AND (“progressive” [tiab] OR “graded” [tiab]).

### Inclusion and exclusion criteria

**Inclusion criteria:**

1. Population: Patients clinically diagnosed with HF according to the European Society of Cardiology criteria (5), with no restriction on HF type.
2. Intervention: The intervention group received Combined endurance and resistance exercise training.
3. Comparison: Endurance exercise training alone without resistance exercise training.
4. Outcomes: Reported at least one of the following: peak VO_2_, QoL, 6MWD, sleep parameters, or depression scores.

**Exclusion criteria:**

1. Non-original research, such as reviews, conference abstracts, case reports, etc.
2. Interventions not focused on the combination of resistance and aerobic exercise.
3. Absence of a clear control group or unclear intervention description.
4. Incomplete data or results that could not be extracted.

## Screening and data extraction

Two independent researchers conducted the initial screening based on titles and abstracts, excluding clearly irrelevant or non-conforming studies. Full-text articles of potentially eligible studies were then retrieved and assessed in detail to ensure compliance with all criteria. Discrepancies were resolved through discussion or consultation with a third reviewer. For the included studies, the two researchers independently extracted data, covering basic study information, participant characteristics, and outcome measures. All extracted data underwent cross-validation to ensure accuracy and consistency, with disagreements settled via discussion or expert consultation.

## Quality assessment

Two independent reviewers assessed the risk of bias in the included randomized controlled trials (RCTs) using the Cochrane Risk of Bias Tool for Randomized Trials (RoB 2). This tool evaluates five domains: randomization process, deviations from intended interventions, missing outcome data, measurement of the outcome, and selection of the reported result. Each domain was judged as having “low risk”, “some concerns”, or “high risk” of bias. The reviewers cross-checked their assessments, and any inconsistencies were resolved through discussion or by consulting a third reviewer.

## Statistical methods

Literature management was carried out using NoteExpress 3.2 software. Data were collected and extracted using Excel 2003. Meta-analysis was conducted using RevMan 5.4.1 software. Heterogeneity among the extracted data was analyzed using the Q-test (*P-*value) and quantified using the I^2^ statistic. A *P-*value > 0.10 or I^2^ ≤ 50% indicated acceptable heterogeneity, warranting the use of a fixed-effects model (FEM) for analysis. Otherwise, a random-effects model (REM) was employed. Pooled effect sizes were expressed as mean difference (MD) or odds ratio (OR) with 95% confidence intervals (CIs). Forest plots were generated for visual representation. The stability of the meta-analysis results was examined using sensitivity analysis. Publication bias was evaluated visually using funnel plots. The statistical significance level was set at *α* = 0.05 (two-tailed).

## Results

### Literature search results

**Figure 1:**
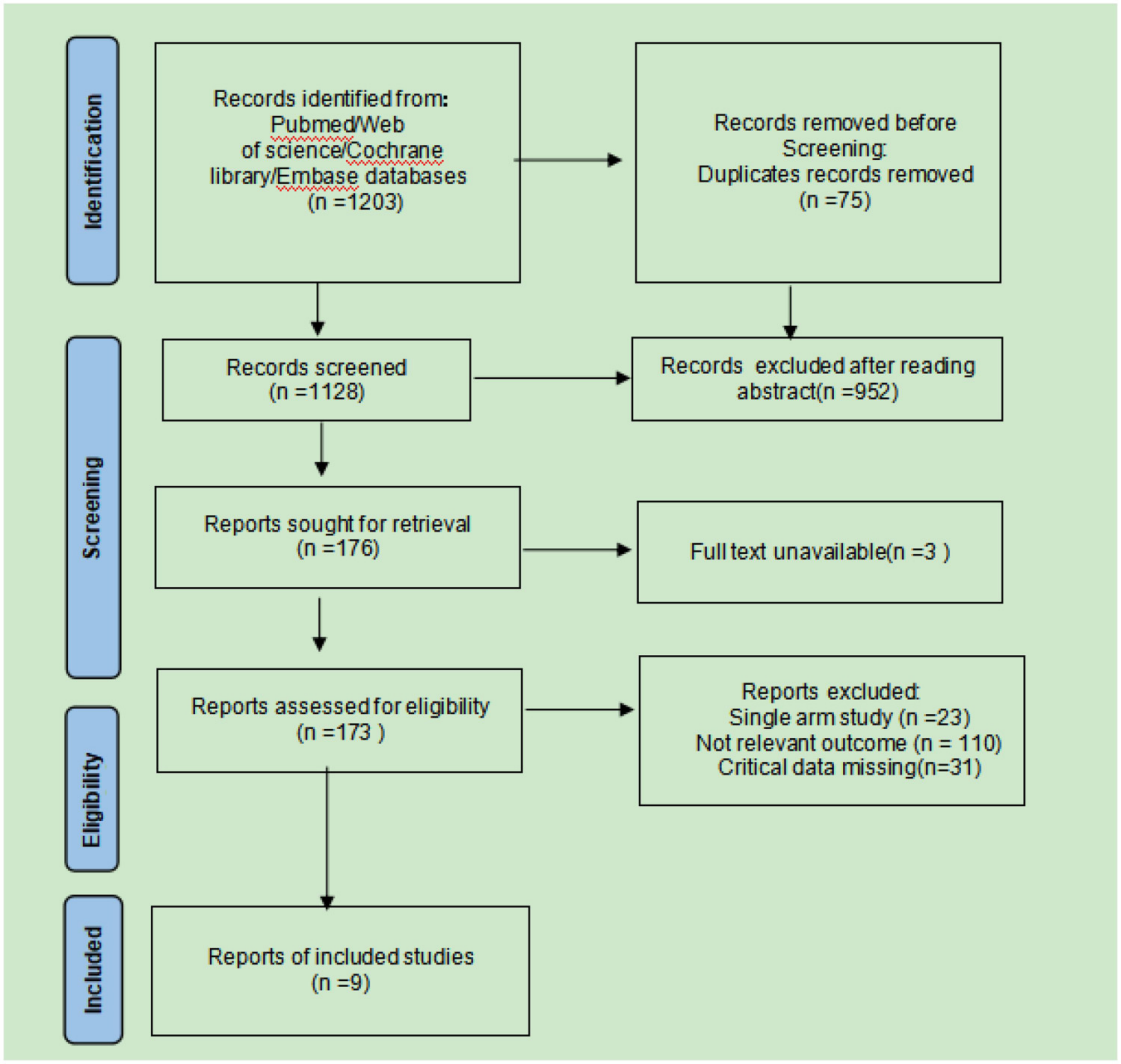
Flow diagram of the literature screening process and results.

The initial search identified 1,203 records from English databases. After removing 75 duplicates using reference management software, 1,128 records remained. Title/abstract screening excluded 952 irrelevant records, leaving 176 for full-text retrieval. Three articles were excluded as full texts were inaccessible. After detailed evaluation of the remaining 173 full-text articles, 164 were excluded for not meeting the inclusion criteria. Ultimately, 9 studies (18–26) were included in the meta-analysis, as shown in Figure 1. The general information of the included studies is presented in Table 1.

**Table 1:** Basic characteristics of the included studies.

Study	Country	Sample		Age		Sex		Outcomes
		Experimental group	Control group	Experimental group	Control group	Experimental group	Control group	
Chrysohoou et al. (18)	Greece	33	39	63 ± 9	56 ± 11	Male: 29 Female: 4	Male: 28 Female: 11	Peak VO_2_, QoL, 6MWD, depression
Ajiboye et al. (19)	Nigeria	28	23	56.1 ± 2.0	51.5 ± 2.6	/	/	Peak VO_2_, 6MWD
Chien et al. (20)	China	24	27	57 ± 16	59 ± 16	Male: 20 Female: 4	Male: 18 Female: 9	QoL, 6MWD, depression
Servantes et al. (21)	Brazil	11	17	50.82 ± 9.45	53.00 ± 8.19	Male: 8 Female: 9	Male: 8 Female: 3	Sleep
Suna et al. (22)	Australia	54	52	61 ± 15	62 ± 13	Male: 46 Female: 8	Male: 36 Female: 16	Sleep
Jolly et al. (23)	Britain	84	85	65.9 ± 12.5	70.0 ± 12.5	Male: 64 Female: 20	Male: 62 Female: 23	QoL, depression
Volterrani et al. (24)	Not mentioned	55	40	63.7 ± 2	62.6 ± 6	Not mentioned		6MWD
Gary et al. (25)	Not mentioned	12	12	59 ± 11	61 ± 10	Male: 7 Female: 5	Male: 5 Female: 7	6MWD
Dracup et al. (26)	Not mentioned	87	86	53.3 ± 12.7	54.6 ± 12.5	–	–	QoL, 6MWD, depression

“–”: The rate calculation in the original text is incorrect.

VO_2_, oxygen consumption; QoL, quality of life; 6MWD, 6-minute walk distance.

### Quality assessment of included studies

**Figure 2:**
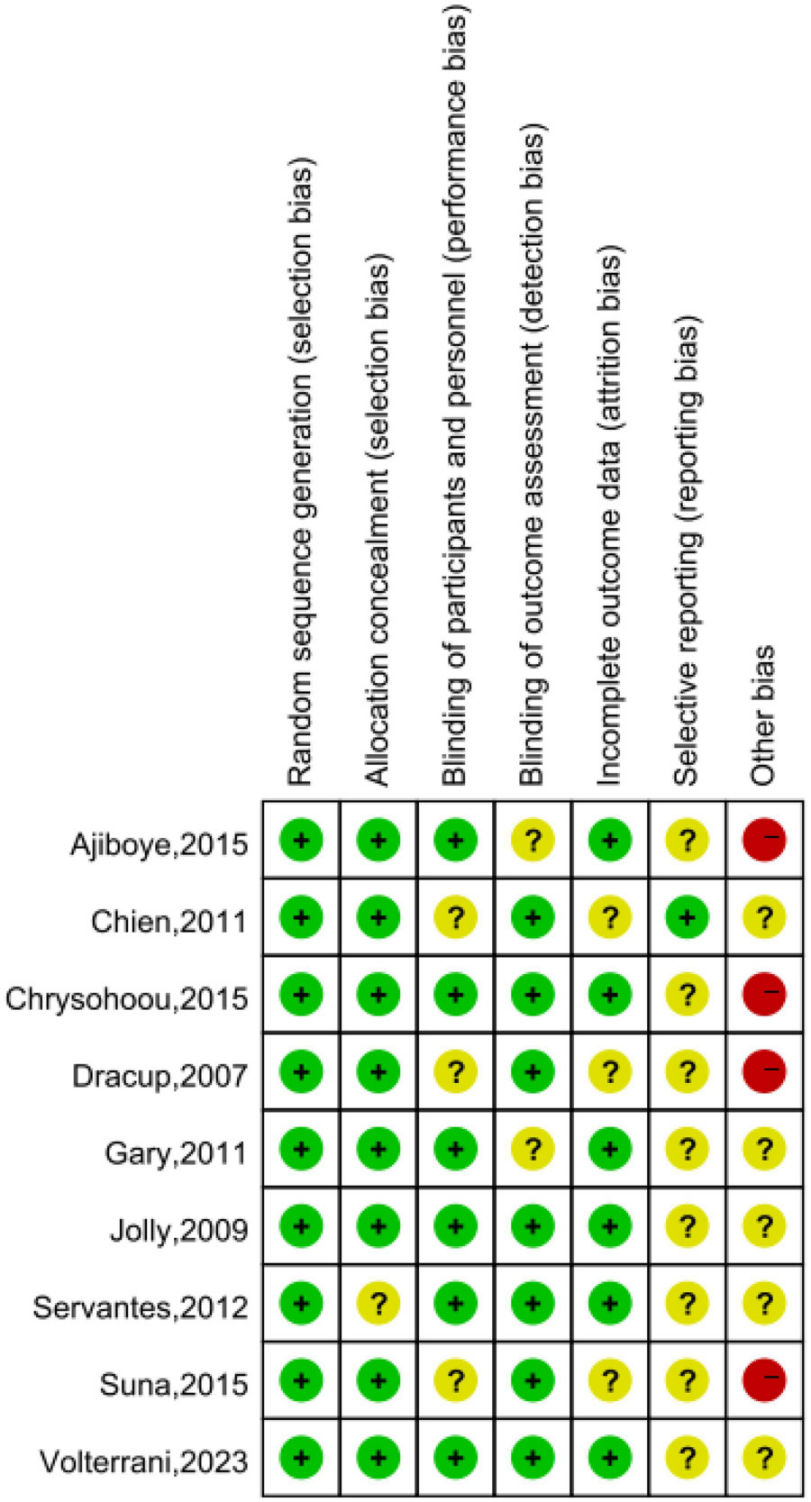
Results of the literature quality assessment.

**Figure 3:**
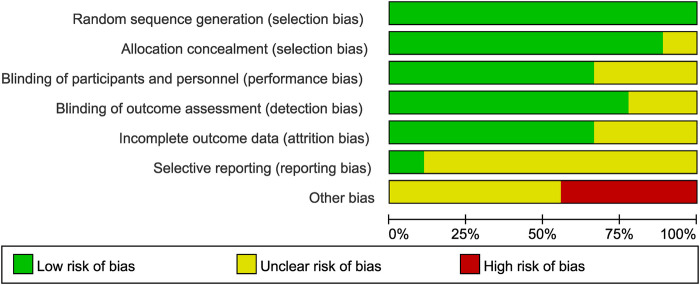
Results of the literature quality assessment.

Quality assessment (Figures 2, 3) indicated that the majority of included studies adhered to RCT design principles. Most studies clearly described their randomization methods, ensuring baseline comparability between groups. Overall, the methodological quality of the included literature was deemed acceptable.

## Results of meta-analysis

### Peak VO_2_

**Figure 4:**
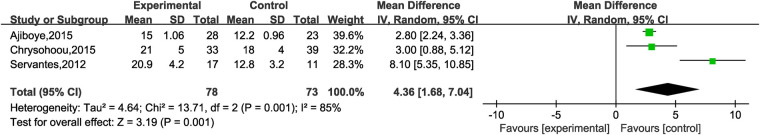
Forest plot for peak oxygen consumption.

Three studies reported peak VO_2_ outcomes, involving 78 patients in the intervention group and 73 in the control group. Significant heterogeneity was observed (I^2^ = 85%, *P* = 0.001); therefore, a REM was used. The pooled analysis showed that peak VO_2_ was significantly higher in the intervention group compared to the control group (MD = 4.36, 95% CI: 1.68–7.04, *P* = 0.001), as shown in Figure 4.

### Qol

**Figure 5:**
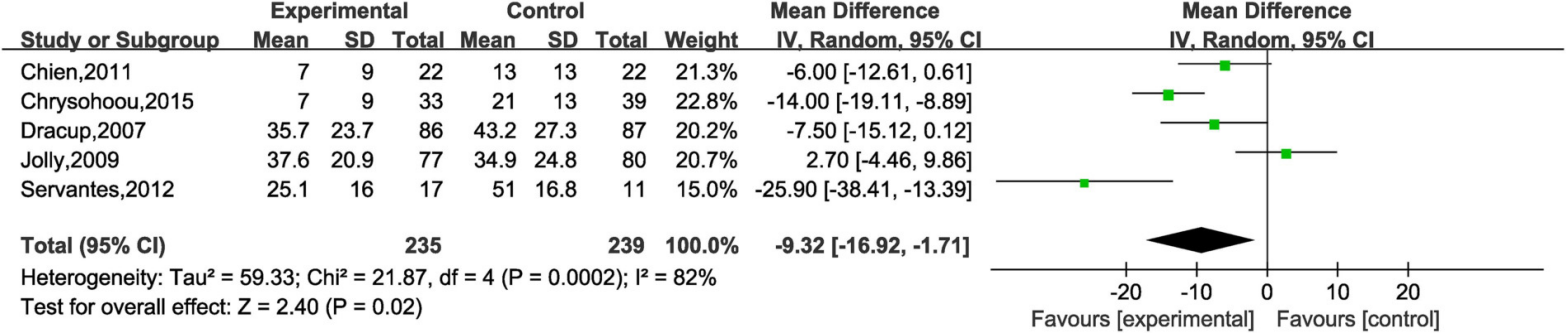
Forest plot for quality of life.

Four studies reported QoL outcomes, involving 235 patients in the intervention group and 239 in the control group. Significant heterogeneity was present (I^2^ = 82%, *P* = 0.0002), leading to the use of a REM. The intervention group demonstrated significantly greater improvement in QoL scores compared to the control group (MD = −9.32, 95% CI: −16.92 to −1.71, *P* = 0.02) (Figure 5).

### Sleep parameters

**Figure 6:**
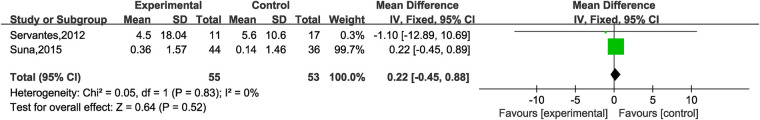
Forest plot for sleep parameters.

Two studies reported sleep outcomes, involving 55 patients in the intervention group and 53 in the control group. No significant heterogeneity was found (I^2^ = 0%, *P* = 0.83); thus, an FEM was applied. The meta-analysis revealed no statistically significant difference in sleep parameters between groups (MD = 0.22, 95% CI: −0.45 to 0.88, *P* = 0.52) (Figure 6).

### Depression scores

**Figure 7:**
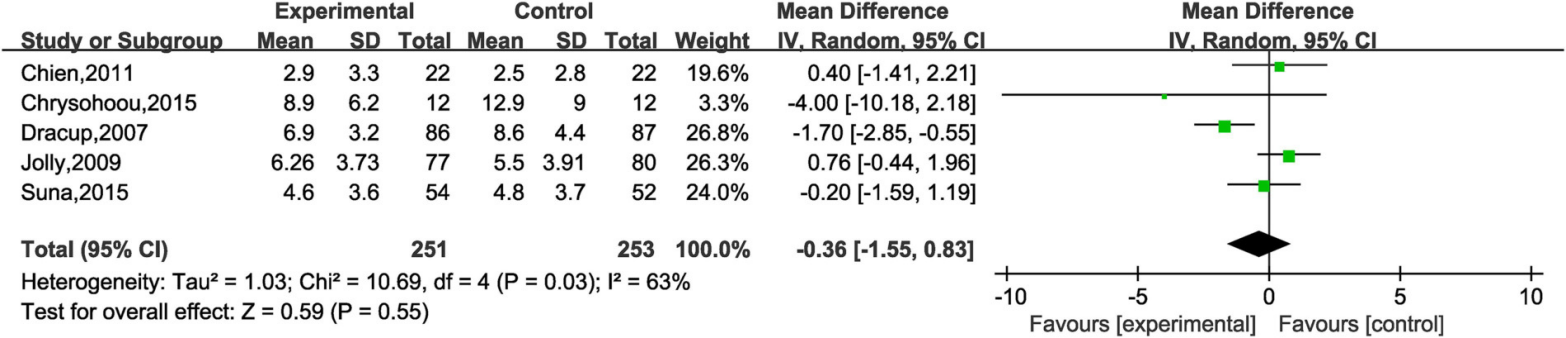
Forest plot for depression levels.

Five studies reported depression scores, involving 251 patients in the intervention group and 253 in the control group. Moderate heterogeneity was observed (I^2^ = 63%, *P* = 0.03), so a REM was used. The analysis found no statistically significant difference in depression levels between groups (MD = −0.36, 95% CI: −1.55 to 0.83, *P* = 0.55), as shown in Figure 7.

### 6MWD

**Figure 8:**
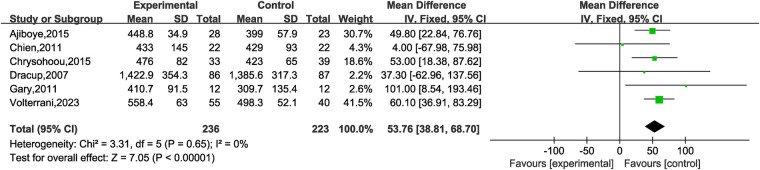
Forest plot for six-minute walk distance.

Six studies reported 6MWD outcomes, involving 236 patients in the intervention group and 223 in the control group. No significant heterogeneity was detected (I^2^ = 0%, *P* = 0.65), warranting an FEM. The 6MWD was significantly greater in the intervention group than that in the control group (MD = 53.76, 95% CI: 38.81–68.70, *P* < 0.0001; Figure 8).

### Sensitivity analysis

**Figure 9:**
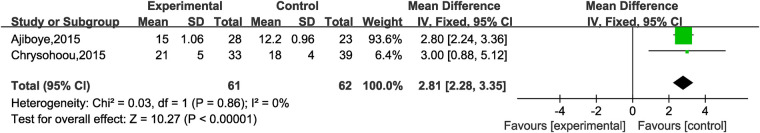
Sensitivity analysis.

A sensitivity analysis was conducted by sequentially excluding each study and recalculating the pooled effect size for the primary outcomes. The results showed that the direction and magnitude of the overall effect size remained stable, and the CIs were not substantially altered, indicating good robustness of the findings (Figure 9).

### Publication bias

**Figure 10:**
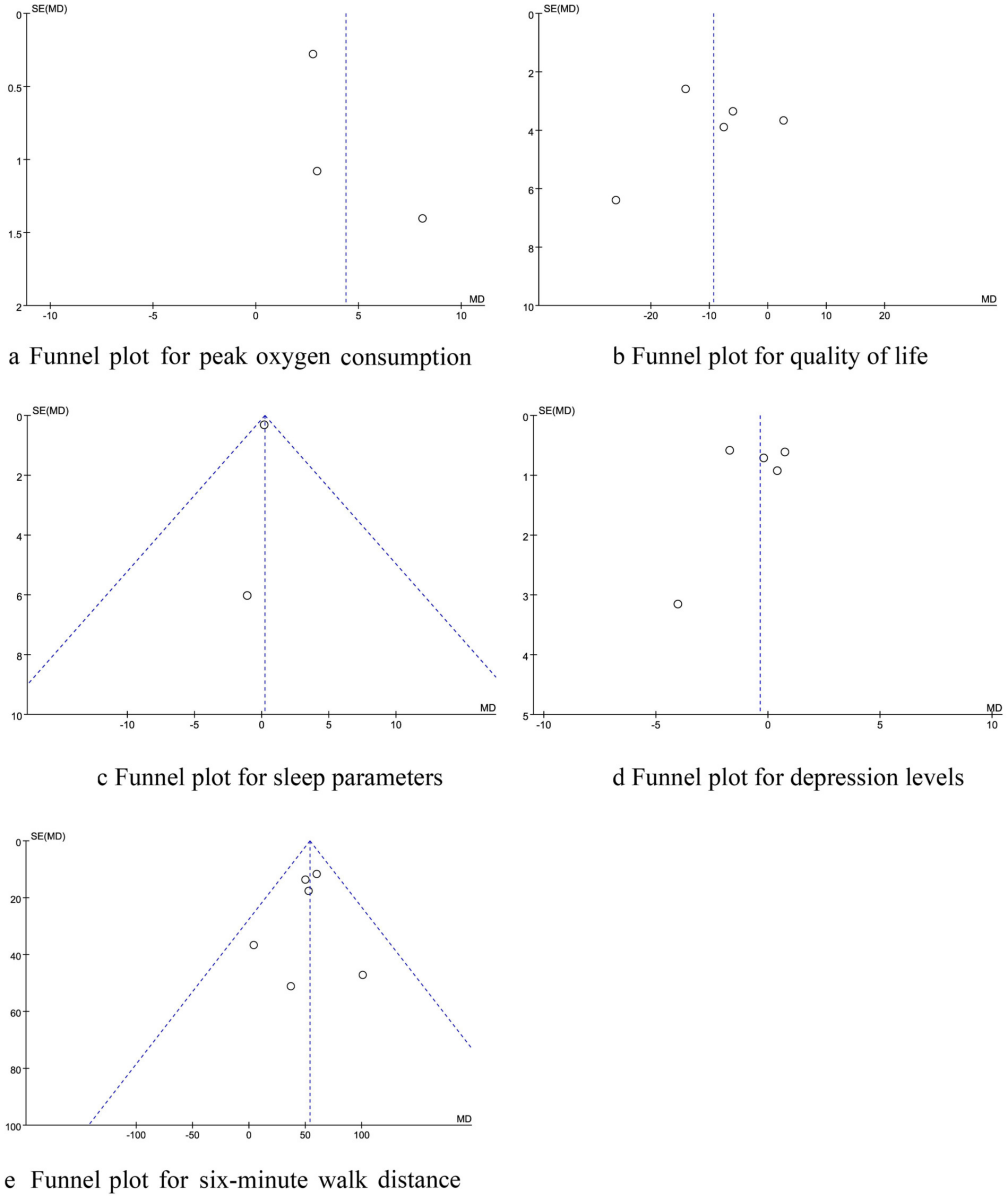
**(a)** funnel plot for peak oxygen consumption. **(b)** Funnel plot for quality of life. **(c)** Funnel plot for sleep parameters. **(d)** Funnel plot for depression levels. **(e)** Funnel plot for six-minute walk distance.

Funnel plots were generated for all outcome measures. Visual inspection suggested approximate symmetry for most plots, indicating a low likelihood of significant publication bias (Figures 10a–e).

## Discussion

Cardiac rehabilitation is a comprehensive medical intervention model encompassing pharmacotherapy, nutritional guidance, exercise prescription, psychological support, and lifestyle modification. Its core objectives are to reduce the risk of recurrence of cardiovascular events, enhance patient QoL, improve clinical prognosis, and ultimately extend healthy life expectancy. In recent years, cardiac rehabilitation services in China have developed rapidly. Rehabilitation facilities and professional teams have expanded, and patients awareness has increased. The prevailing rehabilitation concept has shifted from traditional rest-based approaches to a combination of activity and rest, and the management model has evolved from single-drug prescription to a collaborative “five-prescription” intervention covering medication, exercise, nutrition, psychology and risk factor control. While actively incorporating international advanced experience, China is also exploring a cardiac rehabilitation pathway suited to its national context.

Current guidelines recommend that HF rehabilitation should center on aerobic exercise to improve cardiopulmonary function, supplemented by resistance, flexibility, balance, and respiratory muscle training. The intensity, duration and frequency of exercise should follow the principle of gradual progression, aiming to achieve 3–7 metabolic equivalent hours per week (MET-h/wk), constituting progressive rehabilitation training (27).

Aerobic exercise mainly improves cardiac systolic and diastolic function, increases stroke volume and coronary perfusion, and reduces myocardial oxygen consumption. Resistance exercise exerts milder cardiac load but reduces peripheral afterload and enhances cardiac reserve by improving muscle strength and vascular compliance. Notably, combined endurance and resistance exercise training confers greater advantages in preserving lean body mass and regulating body fat percentage and fat mass compared with endurance exercise alone. However, none of the included studies reported body composition–related outcomes (body fat percentage, fat mass, lean body mass), so this key indicator could not be quantitatively analyzed in the present meta-analysis (28).

HF patients often exhibit markedly reduced exercise tolerance, severely impacting QoL and objectively manifested as decreased peak VO₂ (11). Numerous studies have confirmed that exercise rehabilitation training can effectively increase peak VO₂ in CHF patients (29–31). Underlying mechanisms may involve improved cardiac systolic and diastolic function (32), enhanced vascular endothelial function (33), and optimized skeletal muscle function (34). The latter is considered pivotal for improving exercise tolerance. The research by Esposito et al. (33) demonstrated that an 8-week unilateral knee extension training regimen significantly increased the cross-sectional area of skeletal muscle fibers, mitochondrial density, the ratio of capillaries to muscle fibers, and the proportion of type I muscle fibers in patients with HF with reduced ejection fraction (HFrEF), thereby effectively enhancing skeletal muscle performance.

Cardiopulmonary exercise testing (CPET) is an important means to evaluate cardiopulmonary function and exercise tolerance. Peak VO₂ reflects the maximum oxygen uptake capacity of the body, while the respiratory exchange ratio (RER) characterizes metabolic and ventilatory status during exercise. Research by Kakutani et al. (35) suggests that RER is associated with adverse clinical outcomes in HF. The results of the present study align with evidence showing that regular exercise training following cardiac resynchronization therapy/implantable cardioverter-defibrillator (CRT/ICD) implantation can significantly improve both peak VO₂ and RER compared to device therapy alone, indicating enhanced exercise tolerance. Moreover, ergophysiological assessment using VO₂ at ventilatory threshold 1 (VO₂-VT1) has been increasingly recognized as a more sensitive marker of submaximal endurance capacity than VO₂peak, especially in vulnerable patients with heart failure with reduced ejection fraction (HFrEF) undergoing exercise-based cardiac rehabilitation (36).

Improving QoL has always been an important goal in HF management. Previous studies have shown that poor QoL is a significant predictor of adverse long-term prognosis in HF. Monda et al. (37) found that QoL was closely related to the risk of cardiogenic death in elderly hospitalized patients with HF and served as an independent predictor of all-cause mortality and length of hospital stay related to HF. In this meta-analysis, the rehabilitation intervention group showed significantly greater QoL improvement than the control group.

The six-minute walk test is widely used for functional assessment in elderly and HF populations due to its simple operation, high safety, and exercise intensity that approximates daily activity levels (38). Toukhsati et al. (39) pointed out that successful completion of the test could enhance patients’ confidence in exercise, potentially fostering long-term exercise adherence. Our findings demonstrated that the 6MWD post-rehabilitation training was significantly greater in the intervention group, indicating that the systematic rehabilitation effectively improves exercise endurance in HF patients.

Furthermore, prior research has confirmed that exercise training can ameliorate depressive symptoms in HF. Breazeale et al. (40) highlighted that nearly half of HF patients experienced persistent insomnia, which might impair self-repair mechanisms (41). HF patients frequently suffer from sleep-disordered breathing, insufficient sleep duration, and poor sleep quality (42). However, this meta-analysis did not observe significant improvements in sleep parameters or depression scores. This might be because the mood-enhancing effects of exercise (via neuropsychological pathways) manifest relatively quickly, whereas its impact on sleep pathophysiology intrinsic to HF (such as breathing disorders) may require longer, more intensive intervention. Additionally, the insensitivity of subjective sleep assessment tools might have failed to capture subtle changes.

## Conclusion

Combined endurance and resistance exercise training significant benefits for HF patients, particularly in improving peak VO_2_, QoL, and 6MWD. While no significant effects were observed on sleep or depression in this analysis, the intervention represents a valuable non-pharmacological component of comprehensive HF management. Further high-quality, large-scale RCTs with longer follow-up duration are warranted to confirm these findings and refine optimal training protocols.

## Data Availability

The original contributions presented in the study are included in the article/Supplementary Material, further inquiries can be directed to the corresponding author.
